# Characterizing Movement Patterns of Older Individuals with T2D in Free-Living Environments Using Wearable Accelerometers

**DOI:** 10.3390/jcm12237404

**Published:** 2023-11-29

**Authors:** Tal Yahalom-Peri, Veronika Bogina, Yamit Basson-Shleymovich, Michal Azmon, Tsvi Kuflik, Einat Kodesh, Stefano Volpato, Tali Cukierman-Yaffe

**Affiliations:** 1 Division of Endocrinology, Sheba Medical Center, Ramat-Gan 5266202, Israel; michalaz@ariel.ac.il (M.A.); tcukierm@gmail.com (T.C.-Y.); 2 Epidemiology Department, School of Public Health, Faculty of Health, Tel Aviv University, Tel Aviv 6997801, Israel; basson.yamit@gmail.com; 3 Department of Industrial Engineering, Tel Aviv University, Tel Aviv 6997801, Israel; sveron@gmail.com; 4 Department of Information Systems, University of Haifa, Haifa 3498838, Israel; tsvikak@is.haifa.ac.il; 5 Clalit Health Services, Physical Therapy Clinic, Tel Aviv 6423906, Israel; 6 Faculty of Health Sciences, Ariel University, Ariel 40700, Israel; 7 Department of Physical Therapy, University of Haifa, Haifa 3498838, Israel; ekodesh@univ.haifa.ac.il; 8 Department of Medical Sciences, University of Ferrara, 44121 Ferrara, Italy; stefano.volpato@unife.it

**Keywords:** type 2 diabetes, movement patterns, physical capacity, accelerometer

## Abstract

(1) Background: Type 2 Diabetes (T2D) is associated with reduced muscle mass, strength, and function, leading to frailty. This study aims to analyze the movement patterns (MPs) of older individuals with T2D across varying levels of physical capacity (PC). (2) Methods: A cross-sectional study was conducted among individuals aged 60 or older with T2D. Participants (*n* = 103) were equipped with a blinded continuous glucose monitoring (CGM) system and an activity monitoring device for one week. PC tests were performed at the beginning and end of the week, and participants were categorized into three groups: low PC (LPC), medium PC (MPC), and normal PC (NPC). Group differences in MPs and physical activity were analyzed using non-parametric Kruskal–Wallis tests for both categorical and continuous variables. Dunn post-hoc statistical tests were subsequently carried out for pairwise comparisons. For data analysis, we utilized pandas, a Python-based data analysis tool, and conducted the statistical analyses using the scipy.stats package in Python. The significance level was set at *p* < 0.05. (3) Results: Participants in the LPC group showed lower medio-lateral acceleration and higher vertical and antero-posterior acceleration compared to the NPC group. LPC participants also had higher root mean square values (1.017 m/s^2^). Moreover, the LPC group spent less time performing in moderate to vigorous physical activity (MVPA) and had fewer daily steps than the MPC and NPC groups. (4) Conclusions: The LPC group exhibited distinct movement patterns and lower activity levels compared to the NPC group. This study is the first to characterize the MPs of older individuals with T2D in their free-living environment. Several accelerometer-derived features were identified that could differentiate between PC groups. This novel approach offers a manpower-free alternative to identify physical deterioration and detect low PC in individuals with T2D based on real free-living physical behavior.

## 1. Introduction

Type 2 diabetes (T2D) is a rising epidemic and a global healthcare concern, especially in the older age group. The prevalence of diabetes in older people (age 65–99 years) is projected to increase by about 200% from the estimated number in 2019 and reach 276.2 million by the year 2045 [1]. People with diabetes are at increased risk for frailty due to common diabetes complications [2,3] along with sarcopenia, which is characterized by excessive loss of muscle strength and mass [4]. Both frailty and sarcopenia are known as risk factors for falls [5,6], mobility disability, hospitalization, and even death [1,7]. Preceding the onset of disability and frailty is a long period of deterioration in physical health [8]. Recently, the American Diabetes Association (ADA) published recommendations suggesting older people with diabetes should be screened for cognitive and physical impairments and that medical treatment for people with T2D should be given according to their PC levels and health status [9].

To assess PC, clinicians rely mostly on physical–functional assessment tests which allow quantitative comparisons of performance. Nevertheless, a significant limitation of most of these assessments is their suitability only for clinical environments, requiring supervision. Recent advances in technology have led to an increased use of wearable-sensors that assess functional balance and mobility [10,11]. Accelerometers have been suggested as a quantitative measure of balance and offer a practical and low-cost alternative to force plates which are considered to be the gold standard of balance assessment [12]. These portable, inexpensive, lightweight, and compact sensors allow continuous measurement of movement patterns (MPs) of the wearer both in everyday life and in the clinic [13]. Many studies have used accelerometers for the purpose of gait analysis in order to diagnose and monitor certain diseases [14,15,16,17] or to detect gait patterns in frail older adults [18]. Others have used them to identify a real-time fall [10,19,20] or evaluate the risk of falls through alterations in balance [21,22]. It has even been reported that accelerometer features can better estimate the risk of falls in healthy active community-dwelling older people compared to the use of the timed up and go test (TUG), a common physical-functional mobility test [10]. The current literature has identified a wide range of accelerometer-based features, but there is no consensus regarding the optimal indices to examine from the data obtained [10]. Two commonly used features are the tilt angle and the root mean square (RMS). The tilt angle has been used to detect change in body orientation (such as a fall) or posture and to classify human movement [11,23]. Studies have used the RMS in order to distinguish between responses to different test conditions and between fallers and non-fallers [12,24]. Menz et al. [25] calculated acceleration RMS for all axes (x, y, z) and used it to evaluate age-related differences in walking stability. They found that the magnitude of accelerations at the head and pelvis was generally smaller in older subjects compared to the younger ones. This can be attributed to the reduced walking speed older people tend to adopt as a compensatory strategy to ensure that the head and pelvis remain stable, thereby reducing the likelihood of falls when walking. Kang et al. [11] used a single waist-mounted tri-axial accelerometer in order to classify activities of daily living and to distinguish various active states from resting states in a younger cohort using the RMS and tilt angle. The total successful detection rate of the different activities was approximately 96%. This study provided limited data on MPs in a free-living environment as most data was collected in the laboratory and only one subject was tested in a free-living environment [11].

Characterizing the MPs of older people with diabetes obtained through continuous movement devices may be a first step in identifying those at risk for frailty and disability in a manpower-free objective manner. This might pave the way for implementing interventions that have been shown to prevent further physical deterioration among this population [26,27].

Therefore, this study aimed to characterize MPs of older people with diabetes in varying levels of PC in their ecological environment. 

## 2. Materials and Methods

### 2.1. Participants

In this cross-sectional study, 103 independent adults with T2D over the age of 60 who were randomly selected from diabetes clinics, community centers, and residential care homes were recruited via physicians’ referrals and social media advertisements. Individuals with a significant hearing or visual disability, those who couldn’t walk or perform the physical function tests, and those who were diagnosed with dementia, cognitive impairment, or any major non-diabetes-related illness expected to reduce life expectancy substantially or interfere with study participation were excluded. Individuals who were not able to perform the physical–functional tests for any reason were also excluded from the study.

### 2.2. Study Design

**Figure 1:**
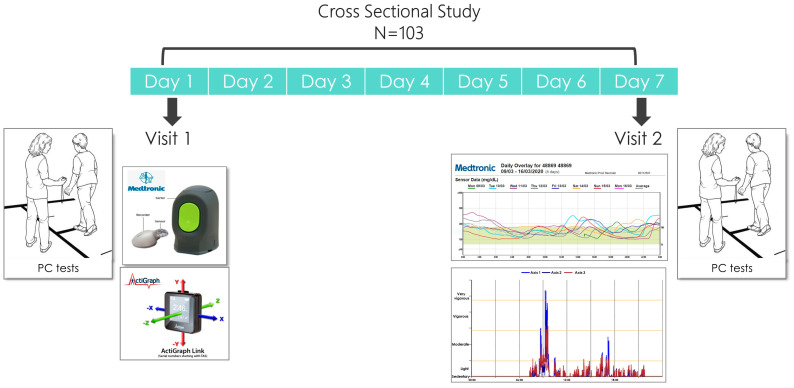
Timeline and description of the seven-day study design that included two visits. On their first visit, participants were connected to a CGM and an accelerometer. One week later, on the second visit, data from both devices was downloaded. On both visits, participants underwent PC (physical capacity) tests.

This study was conducted at the “Center for Successful Aging with Diabetes” at the Sheba Medical Center. Participants visited the clinic twice. On their initial visit, they provided demographic and medical details, completed a questionnaire on physical activity (PA), and underwent various PC tests. These tests encompassed evaluations of aerobic capacity, gait speed, balance, and muscle strength. Participants were then connected to a blinded continuous glucose monitoring (CGM) system (Medtronic iPro™2 and CareLink™, Medtronic, Northridge, CA, USA) and downloaded a compatible application in which they documented glucose levels three to four times a day for calibration purposes. In order to assess MPs and PA behavior, the participants were equipped with an activity recording device (ActiGraph^®^ GT9X; ActiGraph Corp, Pensacola, FL, USA). A week later, the PC measurements were repeated and data from the activity and from the CGM device were downloaded (Figure 1 presents the study’s timeline and description). 

### 2.3. Socio-Demographic Measures

Socio-demographic characteristics, including age, gender, education, marital status, employment status, ethnicity, and smoking status, were collected. Anthropometric measurements, including waist circumference, self-reported weight and height, and calculated body mass index (BMI), were also collected.

### 2.4. Glycemic Control (GC) Status

HbA1c levels were assessed by extracting data from patients’ medical records and the CGM. The CGM system continuously measures and records blood glucose levels at five-minute intervals, with the collected data stored in the sensor and later retrieved for analysis. The data obtained and retrieved from the Medtronic iPro™2 and CareLink™ (Medtronic, Northridge, CA, USA) included: percent of time spent in target range (%TIR, between 70 and 180 mg/dL), time above range (%TAR, >180 mg/dL), time above high range (%TAHR, >250 mg/dL), and time below range (%TBR, <70 mg/dL).

### 2.5. Physical Capacity (PC) Battery

In order to include a wide range of physical abilities and physical capacity domains, well-validated tests with established norms were conducted.

#### 2.5.1. Muscle Strength Assessment

1. The hand grip strength test was used to assess upper body muscular strength [28]. This test was conducted with a dynamometer (Jamar) in a seated position with the patient’s elbow flexed to 90 degrees and their forearm and wrist neutral. An average score (kg) from three repetitions was calculated for the dominant and nondominant hand and compared to the general population according to age and gender. The grip position of the dynamometer was adjusted to each individual’s hand size. Measurements of grip strength taken with the Jamar dynamometer have evidence for good to excellent (r > 0.80) test–retest reproducibility and excellent (r = 0.98) inter-rater reliability [28]. Longitudinal studies confirm that grip strength declines after midlife, with loss accelerating with increasing age and through old age. Grip strength assessment has been shown to have predictive validity, and low values are associated with falls, disability, impaired health-related quality of life, a prolonged length of stay in hospital, and increased mortality [29].
2. The 30 s chair stand (STS) was used to assess lower limb muscle strength [30]. The patient instructions were to stand up from a seated position as many times as possible with arms crossed on the chest for 30 s. Participants were familiarized with the task before the beginning of the test. The number of times within 30 s that the participant could rise to a full stand from a seated position with his back straight and feet flat on the floor “as fast as possible” was counted. The strength of the lower limb muscles has a crucial impact on daily functioning, for example, in movement from a sitting position to a standing position, climbing up stairs, and walking. Failure to perform STS movements efficiently and smoothly may lead to falls [31]. For individuals aged 70–74, a score below 10 signifies a high risk of falling for women, and a score below 12 indicates a high risk for men [32]. To maintain physical independence, a score of 14 or higher is necessary for women, while men require a score of 15 or higher [33].

#### 2.5.2. Aerobic Capacity Assessment

For aerobic capacity, the 6 min walk test (6MWT) [34,35] was conducted. The test measures the distance covered over a total of six minutes on a hard, flat surface. The participants were instructed to walk as fast as they could and were allowed to self-pace and rest as needed as they traversed back and forth along a marked walkway. During the test, participants were discouraged from talking and were notified of each passing two minutes. The distance covered in the 6MWT by healthy adults has been reported to fall within the range of 400 m to 700 m. Additionally, in older adults with heart failure, the 6MWT has been linked to frailty and mortality [36].

#### 2.5.3. Gait Speed Assessment

The 10 m walk test (10MW) was used to determine gait speed [37]. The participants were asked to walk at a “comfortable pace” for a total of 14 m (including two meters for acceleration at the beginning and two meters for deceleration at the end). The score achieved is determined by the elapsed time while the participant walked 10 m. The subject performed the test twice and the average time was reported. Studies have shown that better gait speed is associated with a lower risk for functional decline, hospitalization, and mortality [38] and that a score below 0.8 m/s in the test is a predictor of poor clinical outcomes [39].

#### 2.5.4. Balance Assessment

For the assessment of the participants’ balance, three tests were applied: The timed up and go (TUG) test, the Berg balance scale (BBS), and the four-square step test (FSST).

1. The TUG [40] test examines most mobility skills. The participant is asked to get up from a chair with handles, walk three meters, turn, walk back, and sit down in the shortest possible time. The score is categorized according to the risk of falls and independent walking. The following cut-offs are conventionally used: less than 14 s indicates independent mobility; 15–20 s signifies semi-independent mobility, suggesting a somewhat elevated risk of falls and necessitating further assessment, with the possibility of requiring a walking aid; 20–30 s indicates dependent mobility. Data suggests that the TUG test is a reliable and valid test for quantifying functional mobility and risk for falls that may also be useful in following clinical change over time [41].
2. The BBS [42] test includes 14 tasks which evaluate static and dynamic balance. Each task receives a score of 0 to 4 points depending on the quality and task execution time. The maximum score is 56 points. The scores are dichotomized in the following manner: Scores below 36 indicate impairment with an increased risk of falls, scores between 37- 45 indicate the need for a walking aid in order to walk in a safe manner, and scores above 45 indicate an independent walker without an increased risk of falls. In assessing the risk of fall among the community-dwelling elderly, the TUG and the BBS can be used in combination to increase the diagnostic accuracy of the risk of fall [43].
3. The FSST [44] evaluates dynamic balance at a high functional level and features stepping forward, backwards, left, and right over two 90 cm and 2.5 cm high long sticks that divide the floor into four squares. The subject stands in square 1 facing square 2. The aim is to step as fast as possible into each square with both feet in the following sequence: Square 2, 3, 4, 1, 4, 3, 2, 1 (clockwise to counterclockwise) without touching the sticks. The score is the time required to complete the entire route. Subjects with scores higher than 15 s are associated with a greater risk of falls.

#### 2.5.5. Frailty Assessment

Screening for frailty was performed using the Fried scale [45]. The scale includes five criteria, and pre-frailty is defined as the presence of two components and frailty is defined as the presence of at least three of the following components: (1) unintentional weight loss—loss of 10 lbs/4.5 kg or more in 1 year; (2) self-reported exhaustion/ fatigue; (3) low PA level as assessed by a modified Baecke questionnaire [46]; (4) slow gait speed—less than 0.8 m/s with or without a walking aid; (5) low grip strength relative to gender and body weight.

### 2.6. Allocation to PC Categories

Using the data collected from the PC tests, individuals were then categorized according to their PC status and assigned to one of three groups: the low PC (LPC) group, the medium PC (MPC) group, and the normal PC (NPC) group. Participants were allocated to the LPC group if: their standardized 6MWT or STS or grip score was equal or less than −2 standard deviations or if their BBS score was ≤36, if their TUG score was between 21 and 30, if their FSST score was greater than 15 s, or if they were deemed as frail as assessed by the Fried scale. Participants were allocated to the MPC group if: their standardized 6MWT or STS or grip score was between −2 and −1.5, if their BBS score was between 37 and 45, if their TUG score was between 15 and 20, if their FSST score was between 10.14 and 14.59, or if they were determined to be pre-frail using Fried scale. All other participants whose criteria did not satisfy the previous conditions were allocated to the NPC group [47]. 

### 2.7. Assessment of Movement Patterns (MPs) Using Accelerometers

MPs were measured using an accelerometer (ActiGraph^®^ GT9X; ActiGraph Corp, Pensacola, FL, USA).) worn on the anterior left side of the waist for seven consecutive days. The ActiGraph captures and records high resolution raw acceleration data (i.e., X, Y, and Z coordinates). The following features were derived from the activity device for each patient: timestamp (from which a day, an hour, and a time can be derived), accelerometer recordings collected every 10 ms: (1) Accelerometer X—Mediolateral (ML)—side to side, (2) Accelerometer Y—Vertical (VT)—up down, and (3) Accelerometer Z—Anterior–posterior (AP)—forward and backward (refer to Table A1 in Appendix A). In addition, using the raw data acceleration, the RMS (measured in g-force units where 1 g = 9.8 m/s^2^) and tilt-angle change [11] were calculated. The RMS serves as a measure of acceleration magnitude, computed by taking the average of the square root of the acceleration signals in the x, y, and z directions (x^2^ + y^2^ + z^2^) [22]. The tilt function is employed to identify and assess body posture and orientation (cos^−1^(y)). 

### 2.8. Assessment of Physical Activity (PA) Using Accelerometers

The following indices were extracted from the manufacturer-provided software (ActiLife software version 6.13.4, Pensacola, FL, USA): light PA (LPA), moderate to vigorous PA (MVPA), number of steps, and sedentary time.

### 2.9. Accelerometer Data Integration and Analysis (Figure 2)

The analysis was performed on 815,965,498 data points (accelerometer values); LPC (160,139,810 data points), MPC (323,904,000 data points), and NPC (331,921,688 data points). While all participants were advised to wear the accelerometer continuously for seven days, it was observed that some participants only had recorded data available for a duration of five days. Consequently, the analysis in this study is based on the five-day data records for each participant. We assessed the distribution’s normality using the Kolmogorov–Smirnov test. To ascertain variations in baseline characteristics among the three physical capacity (PC) categories, we conducted non-parametric Kruskal–Wallis tests for both categorical and continuous variables. Dunn post-hoc statistical tests were subsequently carried out for pairwise comparisons. For data analysis, we utilized pandas, a Python-based data analysis tool, and conducted the statistical analyses using the scipy.stats package in Python. The significance level was set at *p* < 0.05.

**Figure 2:**
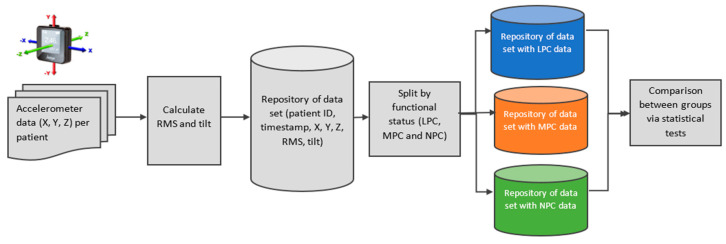
Accelerometer data analysis description and visualization: The process starts with the accelerometers’ data set for 103 patients for one week. The dataset was integrated into a common repository. Three different data sets were extracted according to the patient groups—LPC, MPC, and NPC. At the final stage, the three groups were compared in terms of X, Y, Z, RMS, and tilt a while statistical tests were applied. Note: RMS: root mean square, LPC: low physical capacity, MPC: medium physical capacity, NPC: normal physical capacity.

## 3. Results

### 3.1. Dataset Description

This analysis pertains to data for 103 participants (59.2% male) whose mean age was 71.5 ± 6.9 years and average diabetes duration was 17.1 ± 10.4 years. Twenty participants (19%) met the criteria for LPC, thirty-eight (37%) were classed as MPC, and forty-five (44%) were classed as NPC. Table 1 presents the characteristics of the participants according to their PC category. Diabetes duration (years) was higher in the LPC group compared to the NPC group (*p*-value = 0.004) and in the MPC group compared to the NPC group (*p*-value = 0.009). In addition, compared to the MPC and NPC groups, participants in the LPC group had higher mean glucose levels, spent less time below range (TBR) and in range (TIR), and more time above range (TAR) and above high range (TAHR) (*p*-value < 0.001). 

Table 2 displays the PC test scores and the level of physical activity (PA) for the three groups. It is evident that individuals in the LPC group devoted less time to engaging in moderate to vigorous physical activity (MVPA) in comparison to the MPC and NPC groups and that they took fewer daily steps (*p*-value < 0.001). They also had lower scores on all PC tests except for the STS test. No significant differences in sedentary and light physical activity (LPA) were found between the groups.

### 3.2. Acceleration Patterns of the Three Physical Capacity Categories

Table 3 illustrates the distinctions in acceleration and movement patterns among the three groups. In contrast to the MPC and NPC groups, participants within the LPC group exhibited lower average X-axis acceleration (m/s^2^) (medio-lateral—side to side) but higher Y-axis (m/s^2^) (vertical—up and down) and Z-axis (m/s^2^) (AP—forward and backward) amplitude accelerations. Additionally, they displayed elevated RMS and tilt values.

**Figure 3:**
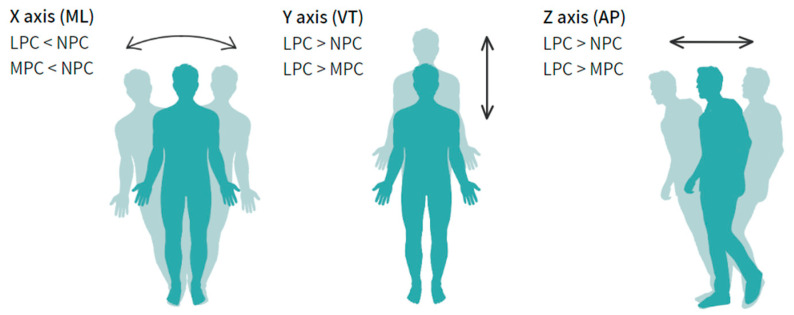
Comparison of x, y, and z acceleration between groups. Only statistically significant comparisons are displayed (*p* < 0.001). Note: ML: mediolateral—side to side, VT: vertical—up and down, AP: anterior–posterior—forward and backward, LPC: low physical capacity; MPC: medium physical capacity, NPC: normal physical capacity.

Figure 3 provides a visual representation of the comparisons between group in the three acceleration axes. Specifically, in the X-axis, individuals in the NPC group exhibited statistically significant higher acceleration signals when compared to the LPC and MPC groups. Conversely, in the Y and Z axes, participants in the LPC group displayed statistically significant higher acceleration signals in contrast to the NPC and MPC groups. 

**Figure 4:**
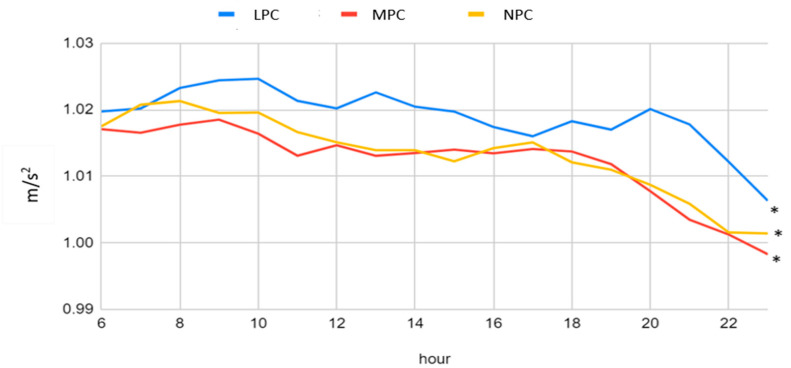
A comparison between the three groups for the median RMS over a five-day period, from 6 AM to 11 PM. * represents *p*-value < 0.001. LPC: low physical capacity, MPC: medium physical capacity, NPC: normal physical capacity, RMS: root mean square.

Significant differences in tilt were identified between the LPC and MPC groups, as well as between the MPC and NPC groups. Figure 4 illustrates that significant differences were also observed in the median RMS acceleration throughout the day among all of the groups (*p*-value < 0.001), with the highest values being evident in the LPC group.

## 4. Discussion

In this study we monitored the movement patterns and physical activity behavior of 103 older people with T2D continuously for one week. The implementation of continuous measurement allowed for real-time identification of movement patterns during the execution of everyday tasks. The findings revealed that participants allocated to the LPC group solely by their PC had lower X and higher Y and Z axis accelerations, as well as higher tilt and RMS values compared to participants in the NPC group. Older individuals with lower PC: i.e., lower scores in agility, balance, aerobic endurance, and muscle strength tests (usually collected through elaborate assessment in the clinic by a physiotherapist) are at higher risks for falls [32,41,42,43,44,48,49]. Our study demonstrated that each PC category is characterized by distinct movement patterns (MPs). Using these MPs may allow early identification of those at high risk for falls and physical deterioration in a remote, manpower-free manner, as opposed to current practice that requires extensive assessment by a health care professional in the clinic. 

This study collected MPs during the execution of everyday tasks. Previous studies used wearable accelerometers mostly in a laboratory setting for gait analysis [14,50] to detect falls [51,52]. Weng et al. [23] aimed to develop a real-time fall detector system via two smartphones worn on the waist and pocket. They reported significant variations in body tilt angles (expressed by posture change) and the RMS (expressed by motion change) when applying a three falling-phase model for the detection of a possible fall. Several studies have highlighted the ability of accelerometer-derived features to predict the risk of falls in older people [10,12]. Among them is the study conducted by Dohney et al. [53]. In this study, 40 adults over the age of 65 were required to stand still for 30 s under four different standing balance conditions while wearing an accelerometer on their lower back. The X and Z acceleration axis signals were used to quantify postural sway in each direction as an indicator for balance. The results showed a significantly higher value for the X axis acceleration signal during two different balance standing conditions in those defined as fallers (participants which had experienced multiple falls or one fall requiring medical attention during the 12 months prior to assessment) compared to non-fallers (*p* < 0.01). Values for the Z axis acceleration signal were also different between fallers and non-fallers, with fallers exhibiting significantly increased sway relative to non-fallers (*p* < 0.05). 

Few researchers have used the accelerometer in a free-living continuous manner similar to our study. Weiss et al. [54] continuously monitored everyday activity over three days to assess fall risk in 71 older adults during community ambulation (i.e., the ability to walk and move around one’s community or neighborhood, navigating and participating in daily activities). The subjects were classified as fallers or non-fallers based on a history of two or more falls and were assessed both in the laboratory and in a free-living environment. They demonstrated that the fallers had significantly higher gait variability in the Y (vertical) axis and lower variability in the X (medio-lateral) axis (*p*-value < 0.02). They suggested that by measuring raw acceleration data during activities of daily living (ADL), those who are at risk to fall can be identified [54]. Another study focusing on fallers and non-fallers, defined based on a comprehensive history of falls, measured the torso accelerations of older patients using a tri-axial accelerometer under four balance conditions. They found a significantly higher acceleration RMS in fallers compared to non-fallers (*p*-value < 0.011) when standing unsupported on a mat with eyes open, and concluded that accelerometer-based measures are potentially an efficient, quantitative alternative tool for balance measurement in older people [12]. 

## 5. Conclusions

To the best of our knowledge, this is the first study attempting to characterize the MPs of older people with T2D in their free-living environment. Through the utilization of wearable accelerometers, we successfully profiled the movement patterns of older individuals across various levels of PC. As the ADA recommends medical treatment for people with T2D according to their PC levels [9], there is an urgent need for effective objective evaluation tools that can detect the early stages of physical deterioration. As most evaluations are conducted in the clinic, require supervision, and are costly and time consuming, this innovative approach might offer a manpower-free alternative way to identify physical deterioration and to detect LPC in people with T2D through free-living ambulation. Larger studies are needed in order to further validate these results, and prospective studies are required to assess the temporality of the relationship. 

### Study Limitations

This study is subject to several limitations that should be acknowledged. Firstly, the cross-sectional design employed does not allow for the establishment of a temporal relationship between the PC level and the MPs. To investigate such a relationship, a prospective study design would be necessary. Secondly, it is important to consider that a portion of the study took place amidst the COVID-19 pandemic, potentially influencing and limiting the participants’ physical activity behavior and MPs. Lastly, to validate and broaden our findings, a larger cohort would be required.

## Figures and Tables

**Table 1:** Comparison between the three PC groups according to demographic, diabetes indices, and glucose levels.

	Total	LPC (*n* = 20)	MPC (*n* = 38)	NPC (*n* = 45)	*p*-Value of the Model (KW)
Gender: Male	61 (59.2%)	10 (50%)	23 (61%)	28 (62%)	0.615
Age	71.5 ± 6.9	71.9 ± 7.4	71.4 ± 6.7	71.3 ± 7.2	0.974
Education (years)	15.4 ± 3.6	13.9 ± 2.6	15.3 ± 3.4	16.2 ± 3.9	0.108
Weight (Kg)	80.4 ± 15.5	79.9 ± 15.5	83.7 ± 16.5	77.9 ± 14.5	0.190
Height (cm)	168.7 ± 9	164.4 ± 7.5	169.9 ± 9.8	169.4 ± 865	0.057
BMI	28.2 ± 4.6	29.3 ± 4.7	29 ± 5.4	27 ± 3.7	0.070
WC (cm)	105.7 ± 11.5	109.9 ± 12.8	106.1 ± 12.4	103.7 ± 9.9	0.170
Falls	24(23%)	5(26.3%)	12(30.6%)	7(15.6%)	0.260
Smoking	10 (9.2%)	4 (17.7%)	2 (5.3%)	4 (9.3%)	0.340
**Diabetes duration (years)**	**17.1 ± 10.4**	**21.2 ± 8.9**	**19.4 ± 11.2**	**12.9 ± 9.1**	**0.0014**
Diabetes complication	96 (93.2%)	19 (95%)	36 (94.7%)	41 (91.1%)	0.760
Severe hypo	18(17.4%)	5(25%)	7(18.4%)	6(13.33%)	0.510
Insulin (%)	89 (86.4%)	19 (95%)	30 (79%)	40 (89%)	0.170
**A1C (%)**	**7.1 ± 1.1**	**7.5 ± 1.2**	**7.1 ± 0.9**	**6.9 ± 1.2**	**0.057**
**Glucose Level (mg/dl)**	**142.7 ± 45.6**	**159.5 ± 53.7**	**141.7 ± 42.2**	**135.7 ± 42.5**	**<0.001**
**TBR (%)**	**1.2 ± 10.8**	**0.9 ± 9.3**	**0.9 ± 9.5**	**1.6 ± 12.6**	**<0.001**
**TIR (%)**	**81.9 ± 38.5**	**71.5 ± 45.1**	**82.5 ± 38**	**86.4 ± 34.3**	**<0.001**
**TAR (%)**	**13.9 ± 34.6**	**20.9 ± 40.7**	**14.4 ± 35.1**	**10 ± 30**	**<0.001**
**TAHR (%)**	**2.9 ± 17**	**6.6 ± 24.9**	**2.2 ± 14.7**	**2 ± 14**	**<0.001**

Numbers are presented as either mean ± SD or *n* (%). Statistically significant rows are bolded. Note: LPC: low physical capacity, MPC: medium physical capacity, NPC: normal physical capacity, KW: Kruskal–Wallis, BMI: body mass index, WC: Waist circumduction, TBR: time below range (>70 mg/dL), TIR: time in range (70–180 mg/dL), TAR: time above range (>180 mg/dL), TAHR: time above high range (>250 mg/dL).

**Table 2:** Comparison between the three PC groups according to physical and functional indices.

	Total	LPC (*n* = 20)	MPC (*n* = 38)	NPC (*n* = 45)	*p*-Value of the Model (KW)
**PA questionnaire—Total score**	**5.3 ± 1.8**	**4.5 ± 1.7**	**5.3 ± 1.7**	**5.8 ± 1.7**	**0.012**
**GRIP, dominant hand (KG)**	**24.9 ± 9**	**18.7 ± 8.3**	**25.7 ± 8.2**	**28 ± 9.5**	**<0.001**
**BERG total score**	**53.9 ± 4.9**	**50.3 ± 7.3**	**54.6 ± 4.6**	**55.1 ± 2.6**	**<0.001**
**FSST (s)**	**10.8 ±3.4**	**14.4 ± 4.8**	**11.4 ± 1.7**	**8.5 ± 1.7**	**<0.001**
**6MWT (m)**	**495.8 ± 111.2**	**376.3 ± 95.1**	**485.2 ± 9**	**557.9 ± 8**	**<0.001**
STS (reps)	13.9 ± 1.5	13.7 ± 1.2	13.9 ± 1.5	13.9 ± 1.7	0.74
**TUG (s)**	**9.2 ± 3.3**	**12.6 ± 5.1**	**9.1 ± 2.3**	**7.6 ± 1.5**	**<0.001**
**10MWT (s)**	**8 ± 1.9**	**9.4 ± 1.9**	**7.9 ± 1.6**	**7.5 ± 1.6**	**<0.001**
**OLS (s)**	**17.8 ± 10.3**	**9.1 ± 8.5**	**17.9 ± 9.6**	**21.1 ± 9.7**	**<0.001**
**3360 turn test (s)**	**5.7 ± 1.8**	**7.4 ± 2.2**	**5.9 ± 1.2**	**4.7 ± 1.2**	**<0.001**
**Pre-frail (%)**		**35**	**10.5**	**0**	
**Frail (%)**		**25**	**0**	**0**	
**Steps (daily mean)**	**4772 ± 2691**	**2535 ± 1411**	**4327 ± 2646**	**4610 ± 1979**	**<0.001**
Sedentary (%)	82.2 ± 7	85.1 ± 6.8	81.9 ± 7.1	81 ± 6.8	0.120
LPA (%)	16.6 ± 6.4	14.4 ± 6.5	16.8 ± 6.6	17.4 ± 6.1	0.247
**MVPA (%)**	**1.2 ± 1.3**	0.4 ± 0.6 *	**1.2 ± 1.3**	**1.5 ± 1.3**	**<0.001**

* Numbers are presented as either mean ± SD or *n* (%). Statistically significant rows are bolded. Note: LPC: low physical capacity, MPC: medium physical capacity, NPC: normal physical capacity, KW: Kruskal–Wallis, PA: physical activity, FSST: four-square step test, 6MWT: 6 min walk test, STS: sit to stand, TUG: timed get up and go, 10MWT: 10 min walk test, OLS: one leg stance, LPA: light physical activity, MVPA: moderate to vigorous physical activity.

**Table 3:** Comparison between the three PC groups according to movement patterns.

LPC (*n* = 20)	MPC (*n* = 38)	NPC (*n* = 45)	LPC (*n* = 20)	*p*-Value of the Model (KW)	Total Median
**X** **(m/s^2^)**	**0.102 (0.57)**	**0.082 (0.63)**	**0.109 (0.61)**	**<0.001**	**0.096 (0.61)**
**Y (m/s^2^)**	**−0.473 (0.76)**	**−0.418 (0.79)**	**−0.438 (0.79)**	**<0.001**	**−0.438 (0.78)**
**Z (m/s^2^)**	**0.234 (1.06)**	**0.141 (1.06)**	**0.172 (1.04)**	**<0.001**	**0.174 (1.06)**
**RMS (m/s^2^)**	**1.017 (0.04)**	**1.009 (0.04)**	**1.011 (0.04)**	**<0.001**	**1.012 (0.04)**
**Tilt (°)**	**1.157(0.52)**	**1.132 (0.49)**	**1.152 (0.52)**	**<0.001**	**1.145 (0.51)**

Numbers are presented as medians (IQR). Statistically significant rows are bolded. Note: LPC: low physical capacity, MPC: medium physical capacity, NPC: normal physical capacity, RMS: root mean square.

## Data Availability

The data that support the findings of this study are available on request from the corresponding author Tali Cukierman-Yaffe.

## Appendix A

**Table A1:** Snapshot of individual patient data, featuring timestamps and corresponding accelerometer signals.

	Timestamp	Accelerometer X	Accelerometer Y	Accelerometer Z
0	12/08/2020 10:55:00.000	0.008	−0.898	0.395
1	12/08/2020 10:55:00.010	0.031	−0.906	0.387
2	12/08/2020 10:55:00.020	0.043	−0.902	0.363
3	12/08/2020 10:55:00.030	0.039	−0.883	0.328
4	12/08/2020 10:55:00.040	0.051	−0.887	0.328
5	12/08/2020 10:55:00.050	0.039	−0.883	0.309
